# Very low risk of lymph node metastasis in Epstein-Barr virus-associated early gastric carcinoma with lymphoid stroma

**DOI:** 10.1186/s12876-020-01422-9

**Published:** 2020-08-17

**Authors:** Yuqing Cheng, Xiaoli Zhou, Kequn Xu, Jin Huang, Qin Huang

**Affiliations:** 1 Department of Pathology of the Affiliated Changzhou Second People’s Hospital of Nanjing Medical University, Changzhou, China; 2 Department of Oncology of the Affiliated Changzhou Second People’s Hospital of Nanjing Medical University, Changzhou, China; 3 Department of Gastroenterology of the Affiliated Changzhou Second People’s Hospital of Nanjing Medical University, Changzhou, China; 4 Department of Pathology and Laboratory Medicine of Veterans Affairs Boston Healthcare System, Harvard Medical School and Brigham and Women’s Hospital, 1400 VFW Parkway, West Roxbury, MA 02132 USA

**Keywords:** Stomach, Carcinoma, Early gastric carcinoma, Gastric carcinoma with lymphoid stroma, Epstein-Barr virus, Lymph node metastasis

## Abstract

**Background:**

Epstein-Barr virus-associated early gastric carcinoma with lymphoid stroma (EBV-GCLS) is a rare variant of early gastric carcinomas. Clinicopathological features of this variant remain obscure, especially in Chinese patients. Therefore, we collected EBV-GCLS cases and studied clinicopathology and prognosis.

**Methods:**

By a retrospective review of 595 consecutive radical gastrectomies for early gastric carcinoma from 2006 to 2018, we identified 8 (1.3%, 8/595) EBV-GCLS cases. Clinicopathologic characteristics were compared between EBV-GCLSs and 109 conventional early gastric carcinomas, which were divided into intramucosal, SM1, and SM2 subgroups. The latter 2 subgroups were classified according to the submucosal invasion depth below or over 500 μm.

**Results:**

All 8 EBV-GCLSs occurred in male patients and invaded deep submucosa (SM2) without lymph node metastasis (LNM), four (50%) of which had synchronous non-gastric malignant tumors (3 gastric gastrointestinal stromal tumors and 1 primary clear cell renal cell carcinoma), and four (50%) arose in the proximal stomach. Compared to conventional early gastric carcinomas, EBV-GCLS was significantly more frequent with SM2 invasion, poor differentiation, and synchronous non-gastric carcinoma tumor, but not with age, gender, macroscopic type, location, size, perineural invasion, lymphovascular invasion, and pathologic stage. In invasion-depth stratified comparisons in the SM2 subgroup, the frequency of LNM in EBV-GCLS was significantly lower than that in conventional early gastric carcinomas (*p* < 0.05) and the 5-year survival rate of patients with EBV-GCLS was better than that with conventional early gastric carcinomas in 3 subgroups (100% vs 91.5, 85.7, 83.9%, respectively), although the differences did not reach a statistically significant level due to the small sample size. Significant differences among 4 subgroups were found in tumor grade, lymphovascular invasion, LNM, pathological stage, and synchronous tumor, but not in age, gender, macroscopic type, tumor size, location, perineural invasion.

**Conclusions:**

Even with poor differentiation and SM2 invasion, EBV-GCLS showed very low risk of LNM and may be a candidate for endoscopic therapy such as endoscopic submucosal dissection.

## Background

According to the World Health Organization statistical data, gastric cancer is the fifth most common cancer and the third leading cause of cancer-related deaths in the world [1]. In China, gastric cancer remains a major health risk, ranking the second in cancer incidence and mortality with very poor prognosis [1]. At present, the only hope for improving gastric cancer patient outcomes is early detection and prompt resection of early gastric carcinoma, which is defined as carcinoma with the invasion depth up to the submucosa, regardless of the lymph node status [2]. With the improvement in endoscopic technology and accumulating operative experience among endoscopists, endoscopic therapy, such as endoscopic submucosal dissection, has become the treatment of choice for early gastric carcinoma in many countries, including China [3].

Gastric carcinoma with lymphoid stroma is a rare variant of gastric carcinomas and associated with Epstein-Barr virus infection in about 80% of cases [4–7]. Epstein-Barr virus-associated early gastric carcinoma with lymphoid stroma (EBV-GCLS) shares some morphologic characteristics with lymphoepithelioma-like carcinoma of the lung, such as poorly differentiated neoplastic cells embedded in the prominent lymphoid stroma [8]. Despite the unfavorable tumor morphology, gastric carcinoma with lymphoid stroma has been reported in previous studies to show low risk of lymph node metastasis (LNM) and good prognosis [6, 7, 9, 10]. However, the clinicopathological features and prognosis of EBV-GCLS, especially in the Chinese patient population, have not been well studied.

In the present study, we aimed to analyze the clinicopathology and prognosis of EBV-GCLS and to compare them with those of invasion-depth stratified conventional early gastric carcinomas without lymphoid stroma or micropapillary components.

## Methods

### Patient selection

Between January 2006 and December 2018, 3039 gastric cancer patients were identified after a search of electronic pathology database for gastric cancer treated with gastrectomy and lymphadenectomy at the Changzhou Second People’s Hospital, which is one of tertiary medical centers in gastric cancer endemic regions in China. Over the study period, we identified 31 early gastric carcinoma patients who were treated with endoscopic submucosal dissection, which has been one of endoscopic resection methods used in our hospital since 2014. Eight of 31 (25.8%) patients also underwent subsequent gastrectomy and lymphadenectomy because of a high suspicion of LNM. Each electronic pathology report was scrutinized for the final diagnosis of early gastric carcinoma on the basis of the World Health Organization definition [2]. Excluded were synchronous gastric carcinomas, gastric stump carcinoma, and lymphoma. Micropapillary adenocarcinoma, an aggressive variant of gastric carcinomas, as described in our previous study, was also eliminated [11]. During the period from January 2010 to December 2012, 109 consecutive cases of conventional early gastric carcinomas with follow-up information were enrolled in the study as the control group for comparison, while 20 cases without follow-up information in this study period were excluded. All enrolled patients underwent D1 or D2 lymphadenectomy and all patients with EBV-GCLS underwent D2 lymphadenectomy. Conventional early gastric carcinomas were grouped into 3 subgroups, according to the invasion depth as intramucosa, superficial (SM1) or deep (SM2) submucosa, which is defined below. All patient private information was deleted and each case was coded with a pathology accession number to protect patient privacy. The study protocol was approved by the Medical Ethics Committee of the Changzhou Second People’s Hospital in China (document number: [2019] KY031–01).

### Clinicopathological investigation

The clinicopathological information on patient age, gender, tumor location, size, and gross feature was retrieved from the electronic pathology archives. The stomach was divided into 5 regions: 1) cardia, defined as the proximal gastric region of about 3 cm below the gastroesophageal junction, 2) fundus, 3) corpus, 4) incisura angularis, and 5) antrum-pylorus. Tumor macroscopic patterns were categorized into 5 groups in accordance with the Paris classification: 1) type 0-I (protruded), 2) type 0-IIa (superficial elevated), 3) type 0-IIb (flat), 4) type 0-IIc (superficial depressed) and 5) 0-III (excavated) [12]. For early gastric carcinoma with mixed macroscopic patterns, the predominant pattern was recorded. The degree of tumor penetration was assessed microscopically and tabulated into 3 subgroups: 1. intramucosa: carcinoma involvement of the lamina propria and muscularis mucosae, 2. SM1: carcinoma penetrating into superficial submucosa (up to 500 μm from the muscularis mucosae), 3. SM2: carcinoma invading into deep submucosa (beyond 500 μm from the muscularis mucosae). The tumor pathologic stage was assigned, according to the 8th edition of the American Joint Committee on Cancer staging manual [13].

All histology slides of each qualified early gastric carcinoma case were reviewed by two experienced study pathologists. The discrepancies on invasion depth and morphologic subtype were minimal and resolved by a joint review for consensus. The histological diagnostic criteria for gastric carcinoma with lymphoid stroma followed those described before [2, 14, 15]: 1) well-demarcated tumor border, 2) poorly differentiated tumor cells with indistinct cytoplasmic border in nest, sheet, anastomosing, or isolated cell growth patterns, 3) dense lymphocytic infiltration in the neoplastic epithelium and throughout the tumor stroma. EBV-GCLS was defined as early gastric carcinoma with lymphoid stroma showing positive nuclear staining for EBV-encoded RNA (EBER) by the chromogenic in situ hybridization test. Lymphovascular invasion, perineural invasion, and tumor budding were also tabulated and analyzed with the methods described in our previous study [16].

### EBV detection

The presence of EBV in early gastric carcinomas was assessed with the EBER chromogenic in situ hybridization test. This was performed manually on unstained tumor sections in 3 μm thickness, using a probe complementary to the EBV-encoded RNA (Zhongshan Jinqiao, Beijing, China). The hybridization signal was detected with an anti-digoxigenin antibody-horseradish peroxidase conjugate and counterstained with hematoxylin. A known EBV-positive nasopharyngeal carcinoma tumor section was included in each run as the positive control. Positive staining was defined as dark brown tumor nuclear staining with negative nuclear signal in surrounding lymphocytes and non-neoplastic cells. A negative control was included in each run by eliminating the EBV-encoded RNA probe.

### Patient survival investigation

In 8 EBV-GCLSs and 109 consecutive cases of conventional early gastric carcinoma over the period from 2010 to 2012, patient survival investigation was carried out via a review of patient’s electronic medical archive, or telephone interview by the authors to patients or patient family members. The number of survival months after radical gastrectomy was calculated from the month of surgical resection to the month of the last follow-up interview or patient death of all causes.

### Statistical analysis

The interobserver variabilities in the invasion depth and morphologic subtype were evaluated by the kappa coefficient test. Clinicopathological features, including patient gender, tumor location, macroscopic pattern, tumor differentiation, invasion depth, lymphovascular invasion, LNM, perineural invasion, pathological stage, synchronous non-gastric carcinoma tumor, and survival were statistically analyzed and compared. Comparisons of categorical variables between groups were determined using the Chi-square or Fisher’s exact test. Continuous values such as age and tumor size were compared between groups using the Student’s t test, least significant difference test, and Mann-Whiney U test. Overall survival rates were estimated with the Kaplan Meier method. A *P* value less than 0.05 was considered statistically significant. All statistical analyses were performed with IBM SPSS Statistics version 19.0 (IBM, Armonk, NY, USA).

## Results

### Clinicopathologic characteristics

The agreements in invasion depth and morphologic subtype between the two observers were almost perfect (κ = 0.92, 0.85, respectively, *p* < 0.05). Gastric carcinoma with lymphoid stroma was found in 11 of 595 (1.8%) qualified early gastric carcinomas, which were identified from 3039 consecutive radical gastrectomies with lymphadenectomies for gastric cancer. Eight of 11 those cases were EBV-positive (1.3%, 8/595) as the study group (Table 1). The control group consisted of 109 conventional early gastric carcinomas (carcinomas without lymphoid stroma nor micropapillary components) with the follow-up data and was further sub-grouped into intramucosal (45.9%, 50/109), SM1 (25.7%, 28/109), SM2 (28.4%, 31/109) subgroups. In the control group, 2 cases were EBV-positive by the EBER in situ hybridization test but did not have lymphoid stroma; 2 additional cases (1.8%, 2/109) were found to have synchronous non-gastric malignant tumors: one as gastrointestinal stromal tumor (pT1N0M0) located in the stomach and another as uterine leiomyoma. The average numbers of retrieved lymph nodes were 22.3, 18.1, 20.5, and 20.7 in the EBV-GCLS group, intramucosal, SM1, and SM2 subgroups of control conventional early gastric carcinomas, respectively.

**Table 1:** Clinicopathological features of EBV-associated early gastric carcinoma with lymphoid stroma

Case	Sex	Age (year)	Macroscopic type	Tumor location	Size (cm)	Proportion of glandular pattern (%)	Invasion depth	Lymphovascular invasion	Lymph node metastasis	Perineural invasion	Synchronous tumor	Follow-up (month)	Status
1	M	66	IIc	Angularis	1.1	5	SM2	0	0	0	GIST in gastric cardia	126	Dead of other disease
2	M	57	IIa + IIc	Antrum	2.5	0	SM2	0	0	0	0	72	Alive without disease
3	M	44	III	Fundus	3.3	45	SM2	0	0	0	0	90	Alive without disease
4	M	60	IIa	Cardia	2	20	SM2	0	0	0	GIST in gastric cardia	60	Alive without disease
5	M	63	III	Cardia	1.8	2	SM2	0	0	0	0	62	Alive without disease
6	M	55	III + IIa	Angularis	3	10	SM2	present	0	0	0	52	Alive without disease
7	M	67	IIc	Antrum	2	20	SM2	0	0	0	ccRCC	42	Alive without disease
8	M	74	IIc	Cardia	1.5	40	SM2	0	0	0	GIST in gastric corpus	18	Alive without disease

*GIST* Gastrointestinal stromal tumor, *ccRCC* Clear cell renal cell carcinoma, 0 Absent

In 23 cases treated only with endoscopic submucosal dissection, gastric carcinoma with lymphoid stroma was not identified. These patients showed a male-to-female ratio of 15:8 with a median age of 64 years. The macroscopic patterns II and III were identified in 20 and 3 cases, respectively. Seventeen cases arose in the gastric antrum or angularis, while 6 cases were in the gastric fundus or corpus. The median size of the tumors was 1.6 cm. The predominant (17/23, 73.9%) morphologic subtype was tubular adenocarcinoma, while papillary adenocarcinoma and poorly cohesive carcinoma were identified in 3 cases each. Submucosa invasion was found in 4 cases (3 to SM1 and 1 to SM2). No lymphovascular invasion was found.

As shown in Table 2, compared to conventional early gastric carcinomas, EBV-GCLS showed no significant differences in patient age, gender, tumor macroscopic pattern, size, lymphovascular invasion, perineural invasion, and pathological stage. However, poor tumor differentiation (100% vs 26, 25, and 35.5% respectively, *p* < 0.01, for intramucosal, SM1, and SM2 subgroups, respectively) and synchronous non-gastric carcinoma tumor (50% vs 2, 3.6, and 0%, respectively) were significantly more frequent in the EBV-GCLS group than in the control groups (*p* < 0.01). EBV-GCLS was commonly (50%, 4/8) discovered the proximal stomach (3 in the cardia and 1 in the fundus). Although the prevalence of SM2 invasion (100% vs 28.4%, *p* < 0.01) was significantly higher in the EBV-GCLS group than in the control group, LNM (0% vs 38.7%, *p* < 0.05) was significantly lower in the former than in the latter in the SM2 subgroup (Table 2).

**Table 2:** Comparison of clinicopathological features between EBV-associated early gastric carcinoma with and without lymphoid stroma

Feature	EBV-associated early gastric carcinoma with lymphoid stroma (*n* = 8)	Conventional early gastric carcinoma without lymphoid stroma (*n* = 109)					
		intramucosa (*n* = 50)	*P*	Superficial submucosa (SM1) (*n* = 28)	*P*	Deep submucosa (SM2) (*n* = 31)	*P*
Age (year)			0.834		0.896		0.875
Median	64.5	62		61		61	
Gender			0.172		0.076		0.168
Male	8 (100.0%)	35 (70.0%)		18 (64.3%)		23 (74.2%)	
Female	0 (0.0%)	15 (30.0%)		10 (35.7%)		8 (25.8%)	
Location			0.066		0.176		0.262
Cardia-fundus	4 (50.0%)	10 (20.0%)		7 (25.0%)		9 (29.0%)	
Corpus-antrum-angularis	4 (50.0%)	40 (80.0%)		21 (75.0%)		22 (71.0%)	
Macroscopic pattern			1.000		1.000		0.686
Non-ulcerated	6 (75.0%)	39 (78.0%)		19 (67.9%)		19 (61.3%)	
Ulcerated	2 (25.0%)	11 (22.0%)		9 (32.1%)		12 (38.7%)	
Tumor size (cm)			0.809		0.864		0.275
Mean ± SD	2.2 ± 0.7	2.1 ± 1.0		2.1 ± 0.7		2.6 ± 1.1	
Tumor differentiation			0.000		0.003		0.001
Well/moderate	0 (0.0%)	37 (74.0%)		21 (75.0%)		20 (64.5%)	
Poorly	8 (100.0%)	13 (26.0%)		7 (25.0%)		11 (35.5%)	
Lymphovascular invasion			0.259		1.000		0.400
Absent	7 (87.5%)	49 (98.0%)		25 (89.3%)		21 (67.7%)	
Present	1 (12.5%)	1 (2.0%)		3 (10.7%)		10 (32.3%)	
Perineural invasion			NA		NA		1.000
Absent	8 (100%)	50 (100%)		28 (100%)		28 (90.3%)	
Present	0 (0%)	0 (0%)		0 (0%)		3 (9.7%)	
Lymph node metastasis			1.000		0.566		0.042
Absent	8 (100%)	48 (96%)		23 (82.1%)		19 (61.3%)	
Present	0 (0%)	2 (4%)		5 (17.9%)		12 (38.7%)	
Pathologic stage			1.000		1.000		0.168
I	8 (100%)	49 (98%)		26 (92.9%)		23 (74.2%)	
II	0 (0%)	1 (2%)		2 (7.1%)		8 (25.8%)	
Synchronous tumor			0.001		0.005		0.001
Absent	4 (50%)	49 (98%)		27 (96.4%)		31 (100%)	
Present	4 (50%)	1 (2%)		1 (3.6%)		0 (0%)	

In comparisons among 4 subgroups, significant differences were found in tumor grade, lymphovascular invasion, LNM, pathological stage, and synchronous tumor (*P* < 0.05), but not in age, gender, macroscopic pattern, tumor size, location, perineural invasion among 4 subgroups.

### Histopathologic features of EBV-associated early gastric carcinoma with lymphoid stroma

**Fig. 1:**
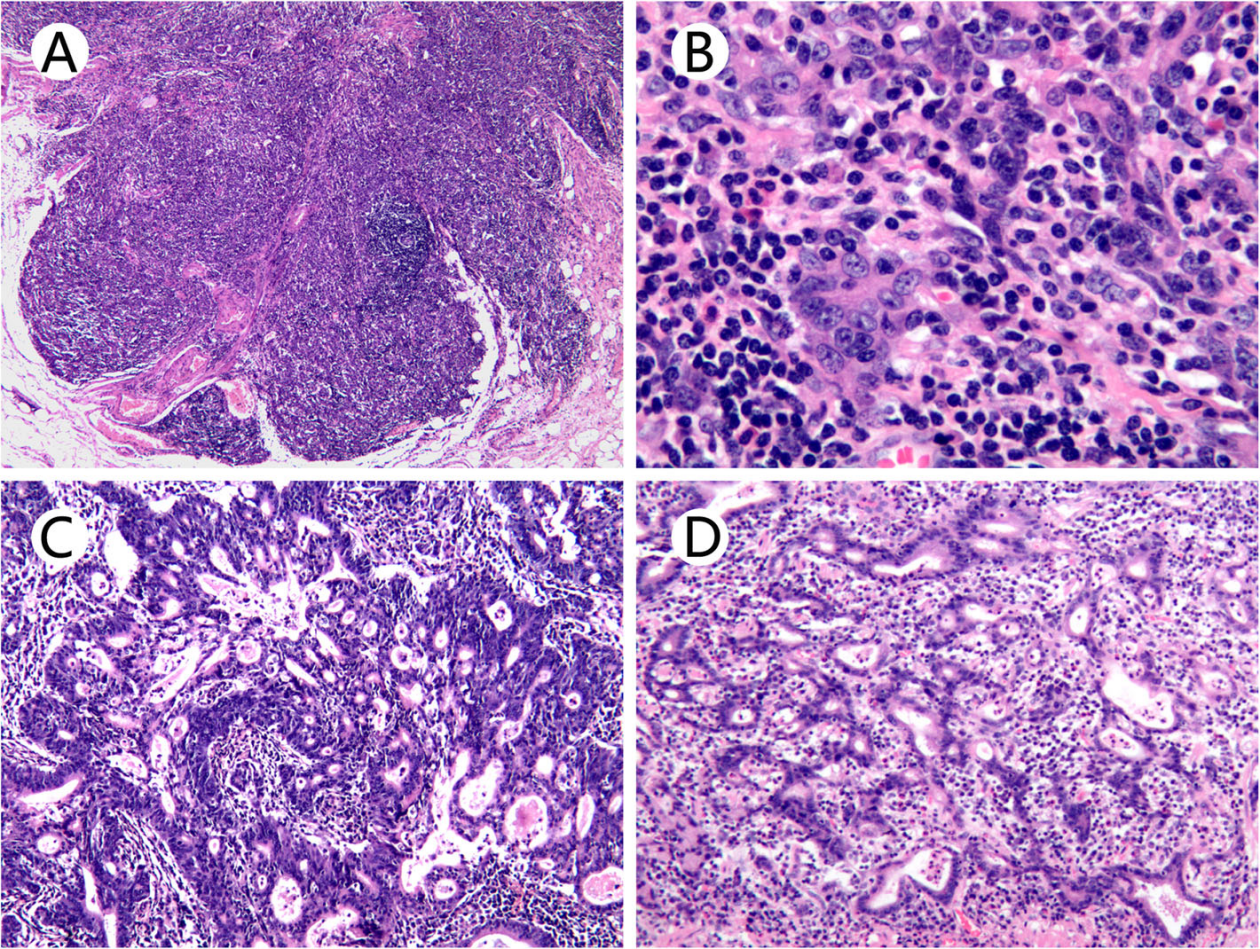
Histopathologic characteristics of Epstein-Barr virus-associated early gastric carcinoma with lymphoid stroma (H&E). **a** The tumor was well-circumscribed with a pushing front and dense intra-tumor lymphocytic infiltration. **b** Tumor cells showed a syncytial growth pattern and poorly formed glandular structures with abundant lymphocytes. **c** The tumor exhibited focally moderately differentiated tubular adenocarcinoma histology with dense lymphocytic infiltration. **d** A lace-like pattern was characterized with anastomosing cords and tubules

**Fig. 2:**
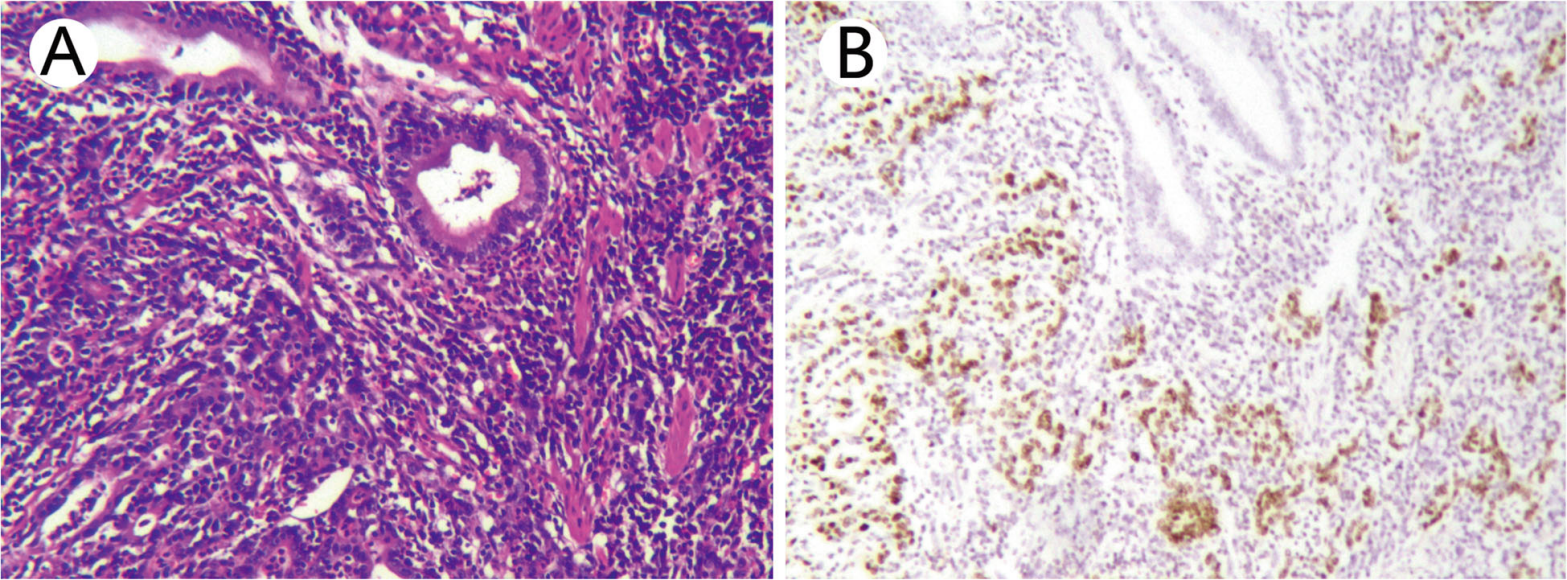
Neoplastic cells, which were inconspicuous in hematoxylin-eosin–stained section (**a**), were highlighted with nuclear-positivity for Epstein-Barr virus-encoded small RNA in situ hybridization, while lymphocytes and non-neoplastic mucosa were negative as internal controls (**b**)

All 8 cases of EBV-GCLS showed an expansile growth pattern with a pushing invasion front at low-power evaluation. The tumor was composed of sheets, nests of, and isolated tumor cells. The anastomosing neoplastic tubular component in the lace-like pattern was present in the lamina propria of 5 (62.5%, 5/8) tumors. Cytologically, poorly differentiated tumor cells appeared to be syncytial with indistinct cell borders, round, oval vesicular nuclei with open chromatin, and distinct nucleoli. Malignant desmoplastic reaction, frequently associated with poorly differentiated conventional carcinoma in the submucosa, was characteristically absent in all 8 EBV-GCLS cases. A minor component of the tubular growth pattern was seen in 7 (87.5%, 7/8) cases, ranging from 2 to 45% in proportion. Intra-tumoral lymphocytic infiltration was prominent in neoplastic epithelial cells and stroma (Fig. 1). The neoplastic epithelial cells showed diffuse positivity in nuclear staining with the EBER in situ hybridization test (Fig. 2). As shown in Table 1, LNM was absent in all 8 cases, and lymphovascular invasion was seen in only 1 (12.5%, 1/8) case. No tumor budding was recognized in any cases. Remarkably, four patients (50%, 4/8) with EBV-GCLS had pathologically confirmed synchronous non-gastric malignant tumors: 3 as gastrointestinal stromal tumor (pT1N0M0) in the stomach and one as clear cell renal cell carcinoma (pT1aN0M0).

### Prognosis

The median follow-up period was 61 months (range, 18 to 126) in all 117 patients of the cohort (the study group: *N* = 8; and the control group: *N* = 109). Patients with EBV-GCLS demonstrated a higher 5-year overall survival rate (100%), compared to those with conventional early gastric carcinomas in 3 subgroups (96.5, 85.7, 83.9% for intramucosal, SM1, and SM2 subgroups, respectively). However, the difference in survival did not reach a statistically significant level because of the small sample sizes.

## Discussion

EBV-GCLS is indeed a very rare (1.3%, 8/595) variant of early gastric carcinomas, but demonstrated in this cohort several important clinicopathologic features as follows: 1. High susceptibility (50%, 4/8) in the gastric cardia and fundus of male patients; 2. Low propensity for lymphovascular invasion (12.5%, 1/8), and the absence of LNM (0%, 0/8); 3. Overwhelming predominance in deep submucosal (SM2) invasion (100%, 8/8); 4. Universally poor differentiation (100%, 8/8); 5. Excessive prevalence (50%, 4/8) for synchronous non-gastric malignant tumors in the stomach (gastrointestinal stroma tumor) and extra-gastric organs (clear cell renal cell carcinoma); and 6. A higher 5-year survival rate, although a significant statistical level was not reached because of the small sample size of study patients. These features, if confirmed with larger samples in the future, may have a significant impact on clinical management of patients with this unusual variant of early gastric carcinomas.

Gastric carcinoma with lymphoid stroma was first report by Watanabe et al in 1976 [17], and its prevalence ranges from 1 to 4% for all gastric carcinomas [5, 18]. The variation in prevalence of gastric carcinoma with lymphoid stroma is much greater in early gastric carcinomas, ranging from 0.6 to 8% [7, 19]. The wide range in prevalence may be due to different study methods for case selection [7, 19]. Several studies in Asia, Europe, and America reveal the prevalence close to 9% for EBV-associated early and advanced gastric carcinomas [19–22]. In our present study, the frequency of EBV infection in gastric carcinoma with lymphoid stroma (72.7%, 8/11) is in agreement with that of previous studies [5–7, 10, 15]. The prevalence of EBV infection in conventional early gastric carcinoma in our study (1.8%, 2/109) was lower than that previously reported [21]. The discrepancy may be related to the exclusion of gastric stump carcinoma and synchronous gastric carcinoma in our cohort, because up to 35% gastric stump carcinomas showed EBV infection and EBV-associated gastric carcinoma often have multiple synchronous gastric carcinomas [20, 23, 24].

As illustrated in the present study, EBV-GCLS demonstrated unique histopathologic patterns characterized by a pushing tumor invasion front, dense intra-tumoral lymphocytic infiltration, poor tumor cell differentiation, high nuclear grade, and sheet, cluster, or nest growth patterns, as reported previously [5, 25, 26]. Tubular-glandular components in various proportions were also observed [5, 15]. Some rare morphologic manifestations, such as epithelioid granulomas, mucinous or signet-ring cell components, and squamous differentiation were not observed in our cohort, but have been described previously [14, 27, 28]. Overall, it is not difficult to make a correct diagnosis for this rare cancer in resection specimens. However, it would be challenging to accurately diagnose this rare early gastric carcinoma in small biopsies, especially in cases with conventional tubular adenocarcinoma showing focal dense lymphocytic infiltrate. Sometimes, dense lymphoid infiltration may lead to a suspicion of mucosa-associated lymphoid tissue lymphoma [4, 17]. However, the intramucosal irregularly anastomosing tubules and cords in a lace-like growth pattern seen in 5 of 8 our cases of EBV-GCLS may be the most important histologic clue for this rare carcinoma in a small biopsy [19, 29, 30]. The cytokeratin immunostaining and EBER in situ hybridization tests would be helpful to rule out the diagnosis of lymphoma by highlighting the existence of neoplastic epithelial cells with characteristic architecture of EBV-GCLS [14, 30].

We showed that EBV-GCLS occurred mainly in male patients and was located primarily in the proximal stomach, poorly differentiated, and invaded deep submucosa (SM2), which parallel to those reported before [6, 7, 19, 26, 31]. Poor tumor differentiation and SM2 invasion in early gastric carcinoma are expected to have a high risk of LNM and should be excluded from endoscopic submucosal dissection [32]. Surprisingly, LNM was absent in all 8 cases of EBV-GCLS in our series. Our findings confirmed the result of previous study by Lim et al, that the frequency of LNM in early gastric carcinoma with lymphoid stroma is significantly lower than that in conventional early gastric carcinoma in T1b stage-matched comparison cases [6]. The very low risk of LNM in EBV-associated gastric carcinoma has been repeatedly reported in both early and advanced gastric carcinomas [9, 14, 18, 19, 33]. The frequency of LNM reported in previous studies of EBV-associated early gastric carcinomas varies from 4.2 to 9.1% [6, 19]. In our series, all 8 EBV-GCLS cases and 63 control cases underwent D2 lymphadenectomy and the average number of retrieved lymph node was 22.3 per case in the EBV-GCLS group and 19.5 per case in the control group, which were lower than that (31 per case) reported previously [34]. The difference may be related to the fact that some cases in our control group had D1, not D2, lymphadenectomy. Moreover, the number of lymph node retrieved may be affected by a variety of factors, such as the surgical and pathologic nodal dissection methods, patient age and immune status, as well as tumor stages, among others [35]. Nevertheless, the results of our study results along with those of previous reports suggest the potential benefits of endoscopic therapy, such as endoscopic submucosal dissection, for EBV-GCLS cases that do not meet the current endoscopic curability indications [6, 19].

The prognosis of patients with gastric carcinoma with lymphoid stroma has been reported to be better than that of conventional gastric carcinoma [6, 9, 10, 36]. In fact, EBV infection has been found to be an independent factor for better overall survival among patients with gastric carcinoma with lymphoid stroma [5]. In our cohort, the overall 5-year survival rate of patients with EBV-GCLS is better than that with conventional gastric carcinoma, irrespective of invasion depth; yet, the difference was not statistically significant, which appear to be related to the small sample size.

One novel, but intriguing finding in our study was the significantly more frequent presence of synchronous non-gastric malignant tumors, especially gastrointestinal stromal tumor, in patients with EBV-GCLS than those with conventional early gastric carcinomas. To the best of our knowledge, only one previous study described the similar finding in gastric carcinoma with lymphoid stroma, in which 2 of 7 cases were associated with gastrointestinal stromal tumor or leiomyoma [14]. The underlying mechanism for the association remains unclear and genetic analysis of more such cases are needed. The implication of this finding is considerable as to appropriate clinical management of patients with EBV-GCLS. Thus, resection strategies for two different tumors in the same patient may need to be carefully evaluated in the decision-making process to balance the pros and cons between endoscopic and surgical resection approaches. For patients with EBV-GCLS and synchronous non-gastric malignant tumors, as shown in Case 5 of our series, it is particularly important to discuss the curability of endoscopic submucosal dissection for EBV-GCLS, especially in the patient who may be unable to tolerate two surgical resections because of poor overall functionality.

There are several limitations to our current investigation. First, any retrospective study inherits selection bias, which was, however, minimized in this study by collecting consecutive cases for this project. Second, although this is the first case series in small case number focused on EBV-GCLS in the Chinese population, the results of our study may not be generalized to other patient populations. Our data require validations by future studies with large samples, especially in other ethnic patient populations.

## Conclusions

Despite the unfavorable pathological features, such as deep submucosal invasion (SM2) and poor tumor differentiation, EBV-GCLS shows very low risk for LNM, which suggests a role of endoscopic therapy such as endoscopic submucosal dissection in the treatment of this rare early gastric carcinoma. Future multicenter studies with lager samples are urgently needed to validate our preliminary findings.

## Data Availability

The datasets used and analyzed during the current study are available from the corresponding author upon reasonable request.

## Abbreviations

EBV-GCLS: Early gastric carcinoma with lymphoid stroma
LNM: Lymph node metastasis
EBV: Epstein-Barr virus
EBER: EBV-encoded RNA
